# Analysis of an evolutionary game of pallet pooling with participation of third-party platform

**DOI:** 10.1371/journal.pone.0256923

**Published:** 2021-10-22

**Authors:** Cuiping Liu, Xinchun Li, Quanlong Liu

**Affiliations:** 1 School of Economics and Management, China University of Mining and Technology, Xuzhou, China; 2 Jiangsu Vocational Institute of Architectural Technology, Xuzhou, China; University of Defence in Belgrade, SERBIA

## Abstract

Pallet pooling has been widely recognized as an important part of the green supply chain. The development of pallet pooling is an essential component for the transformation and upgrade of the logistics industry in China. Pallet pooling can, however, lead to a conflict over potential benefits among the players. One of the main problems of pallet pooling in China is the reasonable benefit allocation mechanism has not been formed. The pallet pooling system (PPS) with participation of the third-party platform (PPSWPTPP) is one of the pilot modes of pallet pooling in China. Based on evolutionary game theory and a penalty mechanism for breach of contract, this paper constructs a tripartite evolutionary game model of the PPSWPTPP. Eight propositions are set in two basic scenarios regarding whether pallet pooling is adapted to the logistics market to study the stability and dynamic evolution process of the players in the PPSWPTPP. Theoretical and numerical simulation results indicate that these will affect the smooth development of the PPS. The suitable setting of penalties and bonuses, the precise estimation of the pooling benefits, the intention intensity of the players to the pallet pooling, and whether to adapt to the logistics market demand are explored.

## 1. Introduction

With the full implementation of "Internet +" and the development of the sharing economy in countries around the world, pallet pooling has been widely recognized as an important part of the green supply chain. As defined in the China National Standard Logistics Terms (GB/T 18354–2006), "the pallet pooling system [PPS] is an organization system in which the interchangeable pallets conforming to the uniform regulations are used to serve many users". In this system, the status of the pallet cargo unit is always maintained until the goods are delivered to the destination, and then the pallets will be returned, after which inspection and proper maintenance will be conducted for the pallets before they enter the system again for reuse.

The countries of Europe, America, Australia, Japan, Korea, and others began the industrial application of pallet pooling in the middle of the 20th century and have witnessed rapid development. In Australia, the CHEP pallet pool, established in 1945, has been used by every major retailer and wholesaler and by the vast majority of producers and suppliers [1]. After World War II, pallets became increasingly important in Europe, which led to the implementation of European pallet standardization by the European Economic Community in 1958 [2]. As of the year 2000 in the United States, more than 93 to 95 percent of unit loads were on pallets [3].

In China, pallet pooling started comparatively late, thought to be no more than ten years (although China’s railway departments had a pallet pooling pilot in 1965, they failed [4]). In recent years, the Chinese government has issued a series of policies and measures to promote the high-quality development of the logistics industry, such as Opinions on Further Promoting Logistics Cost Reduction. The development of pallet pooling is the key point of logistics industry development. On June 5, 2014, Standardization Administration of the People’s Republic of China and Ministry of Commerce of the People’s Republic of China issued Opinions on Accelerating the Standardization of Trade Logistics, and successively selected three batches of 32 cities and 280 key institutions to carry out a pallet pooling pilot in the field of Commerce and Trade logistics. On the basis of the pilot, on December 29, 2017, the Ministry of Commerce and ten departments jointly issued Opinions on Promoting Standard Pallets and Developing Unitized Logistics, which suggests that the development of pallet pooling is an inevitable trend for the transformation and upgrading of the logistics industry in China. It is thus necessary to establish a PPS, as most pallets are limited to internal turnover of enterprises, resulting in a waste of resources [5]. With vigorous promotion by the government, China’s pallet market has achieved a great degree of development, but it has not achieved the expected effect. According to the statistics of the Pallet Committee of China Federation of Logistics and Purchasing, China’s pallet market reached 1.45 billion pieces in 2019, while the delivery rate of unit loads on pallets is only 15%, which is significantly lower than 83% in Europe, 80% in the United States, and 77% in Japan. In China, there are many problems [6, 7] yet to be solved with respect to pallet pooling. One of the main problems is that the effective benefit allocation mechanism has not been formed [4, 8, 9].

The PPS is a multi-agent ecosystem that is affected by several factors, including information asymmetry, external environment change, and insufficient rationality. Further, all the players are limitedly rational, and the players’ decisions are not the results of one selection but are dynamically adjusted and optimized through multiple games and the constant accumulation of experience, which corresponds to the "limited rationality" hypothesis of evolutionary game theory (EGT) and the characteristics of the dynamic optimization of decision making [10]. Thus, an appropriate technique for studying such long-term behavior by pallet pooling players is EGT analysis.

Previous research on pallet pooling has been primarily benefit analysis [5, 11], mode selection [12], standardization and quality control [13], closed-loop supply chain (CLSC), and sustainable development [14] within the short term, and only a few works exist to study pallet pooling in the long term. Further, the mode of "Internet + pallet pooling" is a novel development field, like Uber and Airbnb. Thus, this paper develops a model of PPS with participation of the third-party platform (PPSWPTPP, one of the pilot modes of PPS in China). We intend to contribute knowledge in this area by addressing two key questions: How do the players conduct their game behaviors independently in the PPS? What are the factors that affect the results and stability of game equilibrium? In order to address these questions, we take the ownership of pallet assets as the breakthrough point and utilize evolutionary game theory to study the behavior evolution of all the players in the PPSWPTPP, and explore the influence of relevant parameters under different situations on the evolution dynamics of the three-party pooling system including professional pallet provider (PPP), demanding company (DC), and the third-party information platform (TPIP). The results of our study indicate that the successful operation of the PPSWPTPP is affected by the suitability of the penalty and bonus settings, the precision of the pooling benefit estimation, the intention intensity of the players to the pooling, and the adaptability to the logistics market demand. These results provide guidelines to the managers of the TPIP and to policy makers.

## 2. Literature review

Carrasco, Ponce and Dekker [15] outline the definitions of different reusable articles and analyze three categories: returnable transportation items (RTI), returnable packaging materials, and reusable tools. The pallet is a part of the RTI category, which means the results obtained from that analysis can be extended to the pallet. Three streams of research are of special importance to this study. The first stream of research studies the benefits of pallet pooling, and the second focuses on pallet operation management. The third research stream focuses on players’ decision-making in PPS. All three streams of research will be reviewed briefly below.

### 2.1. Benefits of pallet pooling

Several studies have focused on the economic and environmental benefits of pallet pooling. In particular, the advantages of PPS when compared to open-loop schemes are documented in archival literature. Rosenau et al. [16] found that the financial and environmental benefits can be significantly improved through pallet pooling in comparison to the use of traditional disposable pallets. Hellström and Johansson [17] suggested that the choice of control strategy has a significant impact on investments and operating costs. ZHOU Kang et al. [18] studied the costs of different use methods of the pallet in the railway PPS as well as the economic and social benefits of all parties involved. Here it was found that the economic benefits of the railway PPS are mainly shared by three parties: the pallet demanding company (cargo owner), pallet leasing company (social or railway pallet asset holder), and the railway pallet transport administration authority. The use of a reusable system is economically and environmentally advantageous [19–21]. However, Ray et al. [22] indicated that a pallet rental system may cost more than a pallet purchasing system. Palsson et al. [23] also showed that, in the automotive industry, a single-use packaging system is more attractive from both an economic and environmental perspective. Because RTIs involve a large initial investment [19], depending on the market conditions of each enterprise, pallet pooling is not always the right choice.

### 2.2. Pallet operation management

Scholars have devoted significant effort to the study of mode selection, scheduling, information and traceability when it comes to pallet operation management. Kroon and Vrijens [24] used a stochastic programming method to establish a mathematical model of reverse logistics for reusable transport packaging and planned the storage quantity and transportation route of waste packaging. Breen [25] found that there are several options available for companies to improve the performance of reusable containers. These include aspects such as incentives, moral and legal responsibility, asset management, and outsourcing logistics. LI Tai-Ping [26] found that the problems faced by China in establishing the PPS include standards and quality, the role of government, investors, reasonable layout of service sites, and international cooperation. LUO Jian-Feng et al. [12] believed that the basic factors for the establishment of the PPS in China are the elements of tools, management and support, and organizational forms. Regarding the environmental impacts of pallet management operations, Bilbao et al. [27] proposed a method for choosing pallet management and materials based on the trade-off of cost, durability and environmental impact. Logistics networks entailing pallets are the multi-depot systems in which it is not compulsory for the used empty pallets to return to the issuing depot and can be sent back for reconditioning to any depot in the network [15]. In developing a strategy for pallet service distribution, it is necessary to carefully plan the network infrastructure, the node distribution, and the shipment profile to avoid stockout along the supply chain [28]. For scheduling, Zachariadis et al. [29] studied the route optimization model of vehicles with pallets for satisfying customer demand. Mehrsai et al. [30] studied the real-time scheduling of pallets based on the radial basis function network. Kim, Glock and Kwon [31] pointed out that return lot size and time of RTIs influence deterioration rate of products in the CLSC. Elia and Gnoni [32] proposed a discrete event simulation model to support the management of closed-loop systems for pallets. Bottani et al. [33] conducted optimization of the asset management process in a real CLSC, consisting of a pallet provider, a manufacturer and seven retailers. In combination with new technologies, such as RFID (Radio Frequency Identification), GPS (Global Positioning System), and GIS (Geographic Information System), visual logistics and supply chain management can be realized in the PPS [34–37]. Using a Spanish food company as an example, Alejandro et al. [38] studied the information system for tracking the circulation of shared pallets and delivery units in the whole supply chain. REN Jian-Wei et al. [39] conducted an optimization study on whether RFID tags are adopted for the traceable PPS and found that an RFID system helps to reduce operation cost. Li et al. [40] proposed a pallet pooling information platform (PPIP) using cloud computing in order to overcome the restrictions of PPIP based on traditional IT infrastructure and technology.

### 2.3. Players’ decision-making in PPS

Glock [41] reviewed thirty-three papers related to the decision support models and methods for managing RTI networks, which belonged to four different categories (i.e., packaging system comparison, RTI return forecast, RTI inventory management, and operations management and optimization). This paper falls within the first category, which is the decision-making of players in PPS to choose cooperation or betrayal. Iassinovskaia et al. [42] pointed out that the main factors driving the supply chain members to share pallets are the requirements of the relevant laws and regulations on reduction of the environmental impact and the potential operating benefits. Dubiel [43] distinguished between individual exchanges, multilateral exchanges and pool systems, and pointed out that only after an analysis of the respective enterprise-specific usage can it be decided if a reusable system or a combination of a one-way and a reusable system would be suitable. Grimes-Casey et al. [44] developed a game theory framework for an analysis of the choice between refillable and disposable bottles and pointed out that bottlers only have incentive to use refillable bottles when they are sure that consumer return rates will be reasonably high. Palsson et al. [23] developed an evaluation model for the selection of packaging systems in supply chains from a sustainability perspective. Based on an eco-efficiency goal, Chen Y. J. and Chen H. [45] developed a competitive market model for RTIs, and derived a novel Fenchel core of the cooperative game. Liu, Zhang and Xiao [46] introduced the Shapley model to study the benefit distribution in PPS according to the risk exposure, cost input and level of information sharing of each company.

The results obtained from the analysis of CLSC can be extended to the PPS, which has the characteristics of reverse logistics and CLSC. Li et al. [47] developed an evolutionary game model with a two-echelon CLSC and found that the price of remanufacturing products and government subsidies are critical factors of the development of remanufacturing industry. Based on the Stackelberg game and EGT, Esmaeili, Allameh and Tajvidi [48] studied the short- and long-term behavior of players in a two-echelon CLSC and showed that in the long-term, the remanufacturing process is more profitable for companies compared to a process without remanufacturing. Recently, Shekarian and Flapper [49] studied 230 papers, investigated 196 different structures of CLSCs from a game theory perspective, and pointed out that multiple CLSC channels, reward-penalty mechanisms, different parameters on optimal strategies, players’ behaviors in multiple groups, and multi-periods of CLSC are in need of further research.

In the above studies, certain reference methods and suggestions were provided for the operational practice of PPS, but research on the game-theoretic relationship and benefit coordination among the players of PPS has not attracted significant attention. Therefore, there is a need to study the game-theoretic relationship and benefit coordination mechanism of PPS.

## 3. Model

### 3.1. Model description

**Fig 1:**
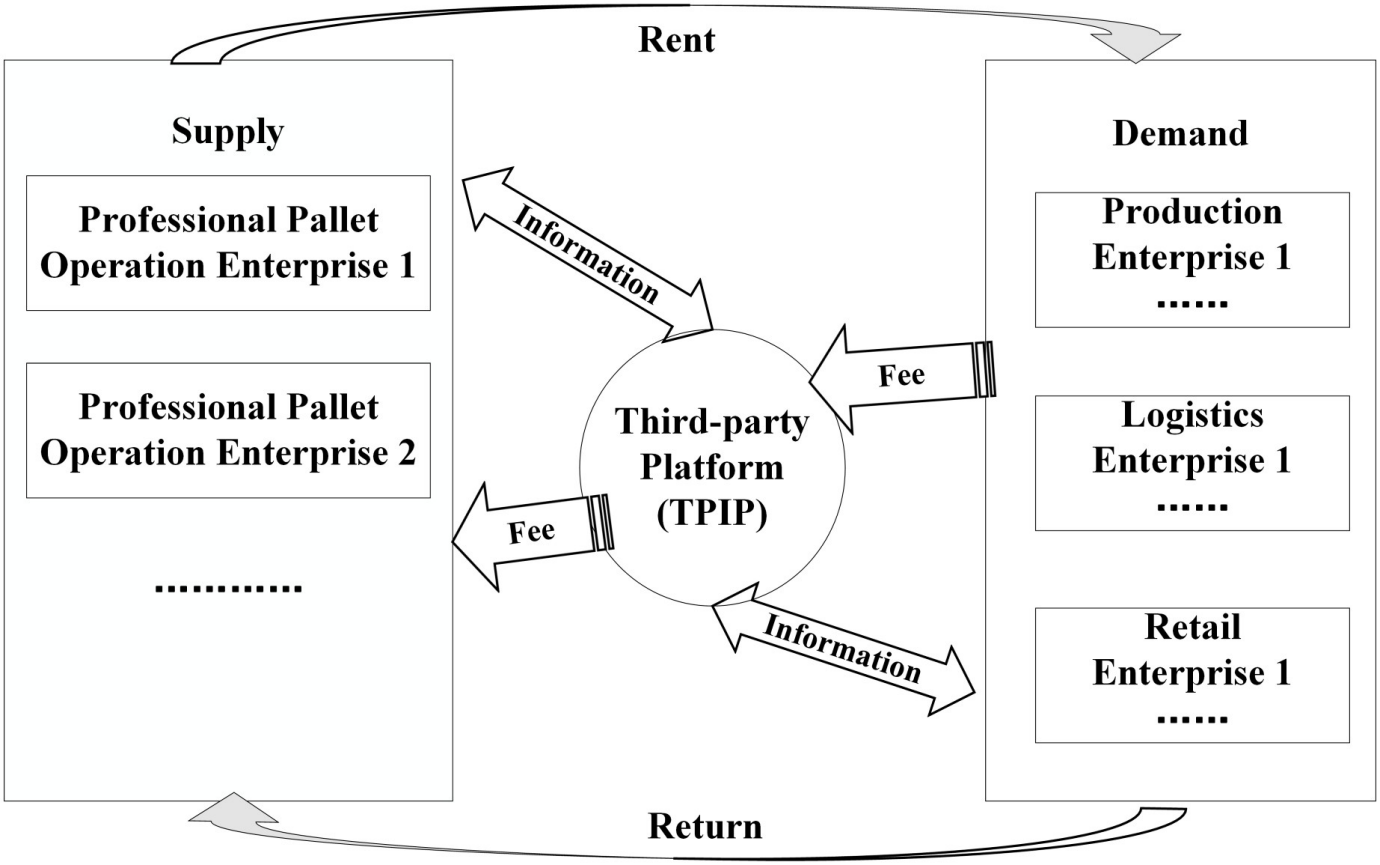
Flow chart of the PPSWPTPP.

The PPSWPTPP refers to the system that has a TPIP that provides information services for pallet pooling, which is independent of the pallet demand and supply sides. The TPIP uses its own complete information network to provide various information services online for pallet demand and supply sides, such as matchmaking, evaluation, supervision and settlement of transactions, and can also integrate service products such as insurance claims, financial support, and mobile APP. The operation process is shown in Fig 1.

This study will be conducted in two stages. First, in the preparation stage, the TPIP invests in the establishment of the information service platform, all the PPPs invest in the pallet and corresponding facilities and equipment, and DCs invest in the corresponding facilities and equipment. Second, in the operation stage, the TPIP provides information services to the supply and demand sides and charges information service fees, the PPPs lease, take back pallets, and collects rent, and the DCs pay the rent; although both the supply and demand sides have joined the platform, in actual operation, they still need to make a decision on whether to rent or lease pallets based on the platform.

### 3.2. Model hypotheses

In order to systematically and comprehensively analyze the evolutionary game model of PPSWPTPP, the following hypotheses are made.

Hypothesis 1: limited rationality. All the players in the system are affected by many limiting factors, such as cognition and environment, when making decisions on pooling behaviors, and they cannot make completely rational decisions, which indicates that pallet pooling is a process in which the players gradually find out better strategies through continuous trial and error and learning, and strategic equilibrium is the result of repeated selection.
Hypothesis 2: random pairing. Although the players have a wide range of choices in the selection of the system partners, we assume that all the players will choose cooperative partners in a random pairing manner.
Hypothesis 3: scene setting. This study considers a PPSWPTPP composed of a PPP, a DC and a TPIP.
Hypothesis 4: basic strategies. We assume that the TPIP has two strategy choices, including supervision and non-supervision; and both DC and PPP can take two strategies, pooling (cooperation) and non-pooling (betrayal). Pooling means that they are willing to participate in the system and actively fulfill their contractual obligations, whereas non-pooling (betrayal) means that they provide pallets by themselves or breach the contractual agreement and give up cooperation.
Hypothesis 5: penalty mechanism for breach of contract. If one player betrays the pooling arrangement, the other player or players may suffer direct losses. In order to promote the smooth development of the pooling arrangement, we set up a penalty mechanism for a breach of contract. When one player chooses the betrayal strategy, the observant player must be compensated as per the penalty mechanism for breach of contract agreed to in advance.
Hypothesis 6: equal game status. We assume that the game status of all the players is equal, and the strategy decisions of all the players does not relate to whether they are active or passive during the pooling.

The expected benefits of the TPIP have network externalities, which are positively related to the quantity of PPPs and DCs and network externality coefficient. The expected benefits of PPPs are mainly from rents, and the expected benefits of DCs are mainly from the reduction of the operation costs of logistics and improvement in the efficiency of logistics through pooling. In order to make the system reasonable and standardized, the TPIP usually takes certain supervision measures for the PPPs and DCs, punish the violators, and reward the compliant parties; strict supervision will increase the operation costs of the TPIP. The notation used in the formulation below is shown in Table 1.

**Table 1:** Notation used in the models.

**TPIP parameters**	
**π**	expected profit under the supervision
**π**′	expected profit of non-supervision
***F***_**1**_	fines to PPP
***F***_**2**_	fines to DC
***G***_**1**_	rewards to the compliant PPP
***G***_**2**_	rewards to the compliant DC
***S***_**1**_	losses caused by PPP
***S***_**2**_	losses caused by DC
**PPP parameters**	
***R***_**1**_	expected benefits of PPP
***C***_**1**_	cost of PPP
**DC parameters**	
***R***_**2**_	expected benefits of DC
***C***_**2**_	cost of DC

Thus, the payment matrix of the tripartite-game of PPSWPTPP is established, as shown in Table 2.

**Table 2:** Game payment matrix of PPSWPTPP.

Strategy		TPP			
		Supervision		Non-supervision	
		DC			
		Cooperation	Betrayal	Cooperation	Betrayal
**PPP**	**Cooperation**	*R*_1_ − *C*_1_ + *G*_1_,	*G*_1_ − *C*_1_,	*R*_1_ − *C*_1_,	−*C*_1_,
		*R*_2_ − *C*_2_ + *G*_2_,	−*F*_1_,	*R*_2_ − *C*_2_,	0,
		*π* − *G*_1_ − *G*_2_	*π* − *G*_1_ + *F*_2_	*π*′	*π*′ − *S*_2_
	**Betrayal**	−*F*_1_,	−*F*_1_,	0,	0,
		*G*_2_ − *C*_2_,	−*F*_2_,	−*C*_2_,	0,
		*π* − *G*_2_ + *F*_1_	*π* + *F*_1_ + *F*_2_	*π*′ − *S*_1_	*π*′ − *S*_1_ − *S*_2_

## 4. Model solving and strategy selection stability analysis

We assume that the probability of the PPP to choose the strategy of cooperation is x (0 ≤ x ≤ 1), and the probability of betrayal is 1 − x; the probability of the DC to choose the strategy of cooperation is y (0 ≤ y ≤ 1), and the probability of betrayal is 1 − y; the probability of the TPIP to choose the strategy of supervision is z (0 ≤ z ≤ 1), and the probability of non-supervision is 1 − z.

### 4.1. Analysis of the strategy stability of players

#### 4.1.1. Strategy stability of the PPP

As per Table 1, the benefit of the PPP to choose the cooperation strategy is

$$\begin{array}{c} U_{c1}=\text{yz}\left(R_{1}-C_{1}+G_{1}\right)+\left(1-y\right)z\left(G_{1}-C_{1}\right)+y\left(1-z\right)\left(R_{1}-C_{1}\right)\\ +\left(1-y\right)\left(1-z\right)\left(-C_{1}\right)=\text{y}R_{1}+zG_{1}-C_{1} \end{array}$$

To choose the betrayal strategy is

$$U_{b1}=\text{yz}\left(-F_{1}\right)+\left(1-y\right)z\left(-F_{1}\right)+y\left(1-z\right)\times 0+\left(1-y\right)\left(1-z\right)\times 0=-zF_{1}$$

The average benefit of the PPP is

$$\bar{U_{1}}=xU_{c1}+\left(1-x\right)U_{b1}$$

and the replicator dynamics equation for selection of the cooperation strategy by the PPP is

$$\begin{array}{cc} F\left(x\right)=\frac{dx}{dt}=x\left(U_{c1}-\bar{U_{1}}\right) & =x\left(1-x\right)\left(U_{c1}-U_{b1}\right)\\ & =x\left(1-x\right)\left(yR_{1}+zG_{1}-C_{1}+zF_{1}\right) \end{array}$$ (1)

From the stability theorem of the replicator dynamics equation [10], we have the following conclusions.

1. when y = [*C*_1_ − (*G*_1_ + *F*_1_)*z*]/*R*_1_, the solution of (1) is *F*(*x*) ≡ 0. This means that the cooperation strategy is a stable strategy for the PPP, and it does not relate to the probability value of the PPP selecting the cooperation strategy.
2. When y ≠ [*C*_1_ − (*G*_1_ + *F*_1_)*z*]/*R*_1_, there are two cases:
  **Case 1**: When 0 < y < {[*C*_1_ − (*G*_1_ + *F*_1_)*z*]/*R*_1_}, we have F′(x)|_*x*=0_ < 0, and F′(x)|_*x*=1_ > 0. Thus, x = 0 is a stable evolution point. In the case where the probability of the DC choosing the cooperation strategy is lower than {[*C*_1_ − (*G*_1_ + *F*_1_)*z*]/*R*_1_}, the PPP selects the betrayal strategy.
  **Case 2**: When {[*C*_1_ − (*G*_1_ + *F*_1_)*z*]/*R*_1_} < *y* < 1, we have F′(x)|_*x*=0_ > 0, and F′(x)|_*x*=1_ < 0. Thus, x = 1 is a stable evolution point. In the case where the probability of the DC choosing the cooperation strategy is higher than {[*C*_1_ − (*G*_1_ + *F*_1_)*z*]/*R*_1_}, the PPP selects the cooperation strategy.

**Fig 2:**
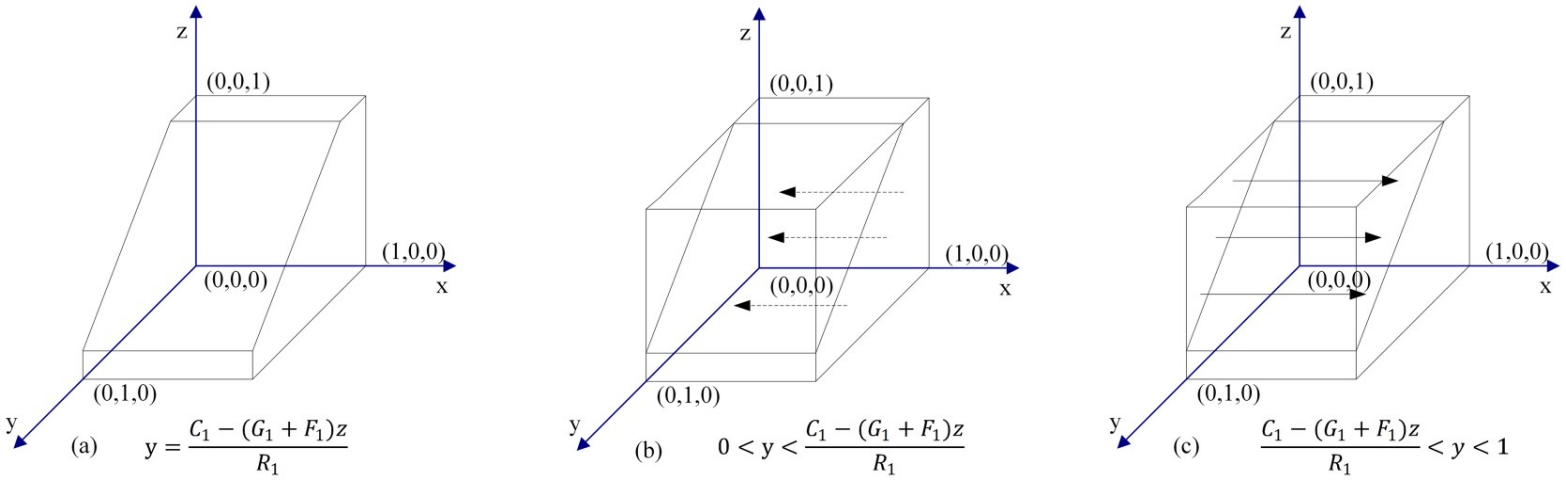
Dynamic evolution chart of PPPs’ decision.

The schematic diagram for dynamic cooperation decision evolution by the PPP is shown in Fig 2.

#### 4.1.2. Strategy stability of the DC and TPIP

Similarly, we obtain the replicator dynamics equations for selection of the cooperation strategy by the DC and the supervision strategy by the TPIP.

The replicator dynamics equation for selection of the cooperation strategy by the DC is

$$\begin{array}{cc} F\left(y\right)=\frac{dy}{dt}=y\left(U_{C2}-\bar{U_{2}}\right) & =y\left(1-y\right)\left(U_{c2}-U_{b2}\right)\\ & =\text{y}\left(1-\text{y}\right)\left(\text{x}R_{2}+zG_{2}-C_{2}+zF_{2}\right) \end{array}$$ (2)

1. When z = (*C*_2_ − *R*_2_*x*)/(*G*_2_ + *F*_2_) or x = [*C*_2_ − (*G*_2_ + *F*_2_)*z*]/*R*_2_, the solution of (2) is *F*(*y*) ≡ 0. This means that the cooperation strategy is a stable strategy for the DC, and it has nothing to do with the probability value of the DC choosing the cooperation strategy.
2. When z ≠ (*C*_2_ − *R*_2_*x*)/(*G*_2_ + *F*_2_) or x ≠ [*C*_2_ − (*G*_2_ + *F*_2_)*z*]/*R*_2_, there are two cases:
  **Case 1**: When 0 < z < {(*C*_2_ − *R*_2_*x*)/(*G*_2_ + *F*_2_)} or 0 < x < {[*C*_2_ − (*G*_2_ + *F*_2_)*z*]/*R*_2_}, we have F′(*y*)|_*y*=0_ < 0, and F′(*y*)|_*y*=1_ > 0. Thus, y = 0 is a stable evolution point. In the case where the probability of the TPIP choosing the supervision strategy is less than {(*C*_2_ − *R*_2_*x*)/(*G*_2_ + *F*_2_)}, or the probability of the PPP choosing the cooperation strategy is less than {[*C*_2_ − (*G*_2_ + *F*_2_)*z*]/*R*_2_}, the betrayal strategy selected by the DC is an evolutionary stable strategy (ESS).
  **Case 2**: When {(*C*_2_ − *R*_2_*x*)/(*G*_2_ + *F*_2_)} < *z* < 1 or {[*C*_2_ − (*G*_2_ + *F*_2_)*z*]/*R*_2_} < x < 1, we have F′(*y*)|_*y*=0_ > 0, and F′(*y*)|_*y*=1_ < 0. Thus, y = 1 is a stable evolution point. In the case where the probability of the TPIP choosing the supervision strategy is greater than {(*C*_2_ − *R*_2_*x*)/(*G*_2_ + *F*_2_)}, or the probability of the PPP choosing the cooperation strategy is greater than {[*C*_2_ − (*G*_2_ + *F*_2_)*z*]/*R*_2_}, the cooperation strategy of the DC is an ESS.

**Fig 3:**
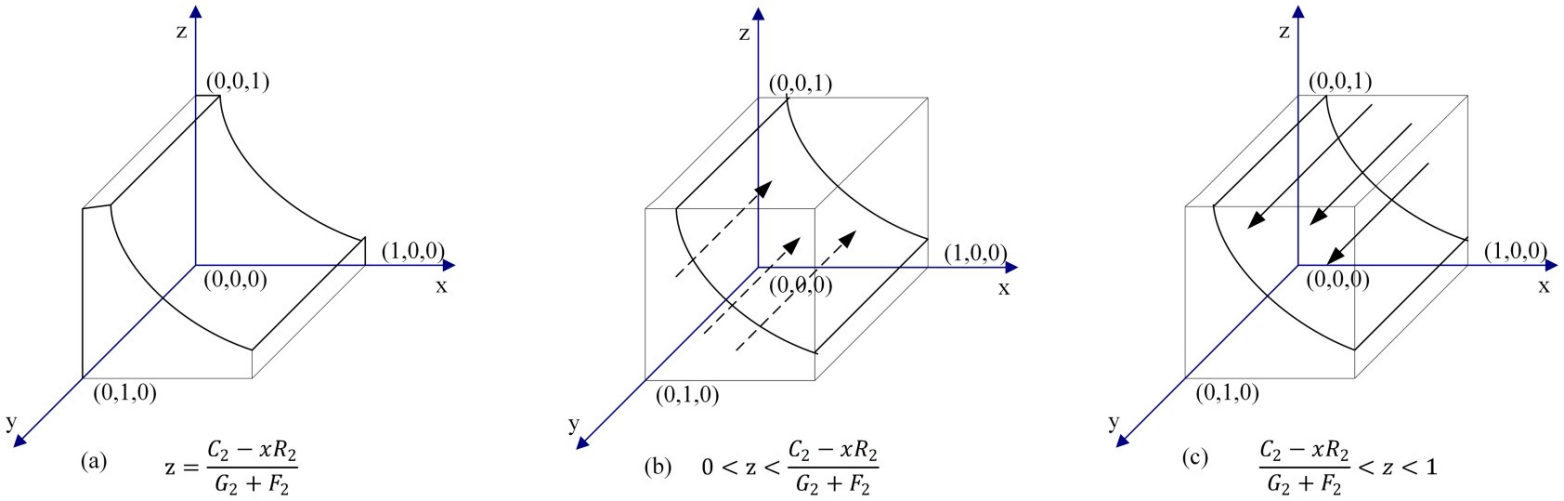
Dynamic evolution chart of DCs’ decision.

The schematic diagram for dynamic cooperation decision evolution by the DC is shown in Fig 3.

The replicator dynamics equation of the TPIP’s selection of the supervision strategy is

$$\begin{array}{cc} F\left(z\right)=\frac{dz}{dt}=z\left(U_{C3}-\bar{U_{3}}\right) & =z\left(1-z\right)\left(U_{C3}-U_{b3}\right)\\ & =z\left(1-z\right)\left[-x\left(G_{1}+F_{1}+S_{1}\right)-y\left(G_{2}+F_{2}+S_{2}\right)+A\right] \end{array}$$ (3)

where *A* = *π* − *π*′ + *F*_1_ + *F*_2_ + *S*_1_ + *S*_2_.

1. When y = [*A* − (*G*_1_ + *F*_1_ + *S*_1_)*x*]/(*G*_2_ + *F*_2_ + *S*_2_), the solution of (3) is *F*(*z*) ≡ 0. This means that the supervision strategy is a stable strategy for the TPIP, and it has nothing to do with the probability value of the TPIP choosing the supervision strategy.
2. When y ≠ [*A* − (*G*_1_ + *F*_1_ + *S*_1_)*x*]/(*G*_2_ + *F*_2_ + *S*_2_), there are two cases:
  **Case 1**: When 0 < y < {[*A* − (*G*_1_ + *F*_1_ + *S*_1_)*x*]/(*G*_2_ + *F*_2_ + *S*_2_)}, we have F′(*z*)|_*z*=0_ > 0, and F′(z)|_*z*=1_ < 0. Thus, z = 1 is a stable evolution point. In the case where the probability of the DC choosing the cooperation strategy is less than {[*A* − (*G*_1_ + *F*_1_ + *S*_1_)*x*]/(*G*_2_ + *F*_2_ + *S*_2_)}, the supervision strategy of the TPIP is an ESS.
  **Case 2**: When {[*A* − (*G*_1_ + *F*_1_ + *S*_1_)*x*]/(*G*_2_ + *F*_2_ + *S*_2_)} < y < 1, we have F′(*z*)|_*z*=0_ < 0, and F′(*z*)|_*z*=1_ > 0. Thus, z = 0 is a stable evolution point. That is, in the case where the probability of the DC choosing the cooperation strategy is greater than {[*A* − (*G*_1_ + *F*_1_ + *S*_1_)*x*]/(*G*_2_ + *F*_2_ + *S*_2_)}, the non-supervision strategy of the TPIP is an ESS.

**Fig 4:**
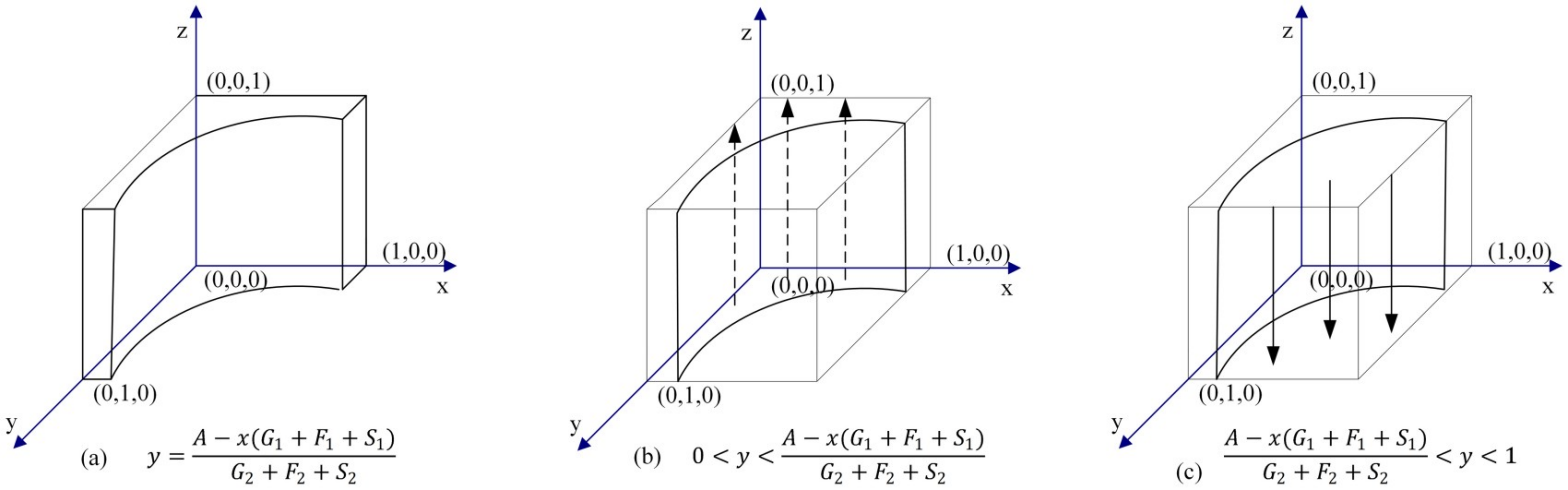
Dynamic evolution chart of TPIPs’ decision.

The schematic diagram for dynamic evolution of the supervision decision of the TPIP is shown in Fig 4.

### 4.2. Third-party strategy selection evolution path and stability analysis

Define

dx/dt=0

dy/dt=0

dz/dt=0

Combining (1), (2) and (3), we obtain the equilibrium points of the PPSWPTPP as follows:

$$\begin{array}{l} E_{1}\left(0,0,0\right),\;E_{2}\left(0,1,0\right),E_{3}\left(0,1,1\right),E_{4}\left(0,0,1\right),E_{5}\left(1,0,0\right),E_{6}\left(1,1,0\right),E_{7}\left(1,0,1\right),E_{8}\left(1,1,1\right),\\ E_{9}\left(0,\frac{C_{1}(G_{2}+F_{2})-C_{2}\left(G_{1}+F_{1}\right)}{R_{1}\;\left(G_{2}+F_{2}\right)},\frac{C_{2}}{G_{2}+F_{2}}\right),E_{10}\left(1,\frac{C_{1}(G_{2}+F_{2})-\left(G_{1}+F_{1}\right)\left(C_{2}-R_{2}\right)}{R_{1}\;\left(G_{2}+F_{2}\right)},\frac{C_{2}-R_{2}}{G_{2}+F_{2}}\right),\\ E_{11}\left(\frac{C_{2}(G_{1}+F_{1})-C_{1}(G_{2}+F_{2})}{R_{2}(G_{1}+F_{1})},0,\frac{C_{1}}{G_{1}+F_{1}}\right),E_{12}\left(\frac{C_{2}(G_{1}+F_{1})-(G_{2}+F_{2})(C_{1}-R_{1})}{R_{2}(G_{1}+F_{1})},1,\frac{C_{1}-R_{1}}{G_{1}+F_{1}}\right),E_{13}\left(\frac{C_{2}}{R_{2}},\frac{C_{1}}{R_{1}},0\right)\\ E_{14}\left(\frac{C_{2}-\left(G_{2}+F_{2}\right)}{R_{2}},\frac{C_{1}-\left(G_{1}+F_{1}\right)}{R_{1}},1\right),E_{15}\left(x^{*},y^{*},z^{*}\right) \end{array}$$

where

$${\text{x}}^{*}=\frac{\left(G_{2}+F_{2}+S_{2}\right)\left[C_{2}\left(G_{1}+F_{1}\right)-C_{1}\left(G_{2}+F_{2}\right)\right]+R_{1}\left(G_{2}+F_{2}\right)A}{R_{1}\left(G_{2}+F_{2}\right)\left(G_{1}+F_{1}+S_{1}\right)+R_{2}\left(G_{1}+F_{1}\right)\left(G_{2}+F_{2}+S_{2}\right)}$$

$$y^{*}=\frac{\left(G_{1}+F_{1}+S_{1}\right)\left[C_{1}\left(G_{2}+F_{2}\right)-C_{2}\left(G_{1}+F_{1}\right)\right]+R_{2}\left(G_{1}+F_{1}\right)A}{R_{1}\left(G_{2}+F_{2}\right)\left(G_{1}+F_{1}+S_{1}\right)+R_{2}\left(G_{1}+F_{1}\right)\left(G_{2}+F_{2}+S_{2}\right)}$$

$$z^{*}=\frac{R_{1}C_{2}\left(G_{1}+F_{1}+S_{1}\right)+R_{2}C_{1}\left(G_{2}+F_{2}+S_{2}\right)-R_{1}R_{2}A}{R_{1}\left(G_{2}+F_{2}\right)\left(G_{1}+F_{1}+S_{1}\right)+R_{2}\left(G_{1}+F_{1}\right)\left(G_{2}+F_{2}+S_{2}\right)}$$

As per evolutionary game theory, in the stable evolution strategy among the players, only the asymptotic stability of the pure equilibrium points (that is, *E*_1_~*E*_8_) must be explored, without analyzing the stability of the mixed equilibrium points [50, 51].

Combining Eqs (1), (2) and (3), the Jacobian matrix J of the system is

$$\begin{array}{c} J=\left[\begin{array}{ccc} \frac{\partial F\left(x\right)}{\partial x} & \frac{\partial F\left(x\right)}{\partial y} & \frac{\partial F\left(x\right)}{\partial z}\\ \frac{\partial F\left(y\right)}{\partial x} & \frac{\partial F\left(y\right)}{\partial y} & \frac{\partial F\left(y\right)}{\partial z}\\ \frac{\partial F\left(z\right)}{\partial x} & \frac{\partial F\left(z\right)}{\partial y} & \frac{\partial F\left(z\right)}{\partial z} \end{array}\right]\\ =\left[\begin{array}{ccc} \left(1-2x\right)\left(yR_{1}+zG_{1}-C_{1}+zF_{1}\right) & x\left(1-x\right)R_{1} & x\left(1-x\right)\left(G_{1}+F_{1}\right)\\ y\left(1-y\right)R_{2} & \left(1-2y\right)\left(xR_{2}+zG_{2}-C_{2}+zF_{2}\right) & y\left(1-y\right)\left(G_{2}+F_{2}\right)\\ -z\left(1-z\right)\left(G_{1}+F_{1}+S_{1}\right) & -z\left(1-z\right)\left(G_{2}+F_{2}+S_{2}\right) & B \end{array}\right] \end{array}$$ (4)

where

$$B=\left(1-2z\right)\left[-x\left(G_{1}+F_{1}+S_{1}\right)-y\left(G_{2}+F_{2}+S_{2}\right)+A\right]$$

Putting 8 equilibrium points (*E*_1_~*E*_8_) into (4), we obtain the eigenvalues of the Jacobian matrixes of the system as shown in Table 3.

**Table 3:** Eigenvalues of Jacobian matrix of PPSWPTPP.

Equilibrium Point	Eigenvalue λ_1_	Eigenvalue λ_2_	Eigenvalue λ_3_
***E***_**1**_ **(0,0,0)**	−*C*_1_	−*C*_2_	*A*
***E***_**2**_ **(0,1,0)**	*R*_1_ − *C*_1_	*C*_2_	*π* − *π*′ − *G*_2_ + *F*_1_ + *S*_1_
***E***_**3**_ **(0,1,1)**	*R*_1_ + *G*_1_ − *C*_1_ + *F*_1_	−(*G*_2_ − *C*_2_ + *F*_2_)	−(*π* − *π*′ − *G*_2_ + *F*_1_ + *S*_1_)
***E***_**4**_ **(0,0,1)**	*G*_1_ − *C*_1_ + *F*_1_	*G*_2_ − *C*_2_ + *F*_2_	−*A*
***E***_**5**_ **(1,0,0)**	*C*_1_	*R*_2_ − *C*_2_	*π* − *π*′ − *G*_2_ + *F*_2_ + *S*_2_
***E***_**6**_ **(1,1,0)**	−(*R*_1_ − *C*_1_)	−(*R*_2_ − *C*_2_)	*π* − *π*′ − *G*_1_ − *G*_2_
***E***_**7**_ **(1,0,1)**	−(*G*_1_ − *C*_1_ + *F*_1_)	*R*_2_ + *G*_2_ − *C*_2_ + *F*_2_	−(*π* − *π*′ − *G*_1_ + *F*_2_ + *S*_2_)
***E***_**8**_ **(1,1,1)**	−(*R*_1_ + *G*_1_ − *C*_1_ + *F*_1_)	−(*R*_2_ + *G*_2_ − *C*_2_ + *F*_2_)	−(*π* − *π*′ − *G*_1_ − *G*_2_)

As per system dynamics theory, the zero solution of the linear homogeneous constant coefficient system is a necessary and sufficient condition for the asymptotic stability point. The eigenvalues of the constant coefficient matrixes are all negative [52, 53], that is, the eigenvalues of the Jacobian matrices corresponding to the evolutionary stable points are all non-positive. In order to analyze the Eigenvalue symbol of the Jacobian matrices corresponding to different equilibrium points, we assume that (1) {*F*_1_, *F*_2_} > *max*{*G*_1_, *G*_2_}, that is, the fines imposed by the TPIP on the violators are greater than the bonuses for the compliant parties. Further, (2) A > 0, that is *π* + *F*_1_ + *F*_2_ + *S*_1_ + *S*_2_ > *π*′ TPIP’s expected profit in the case of supervision plus the losses caused by PPP&DC plus fines to PPP&DC is greater than the expected profit of the TPIP in the case of non-supervision. To analyze the stability of the PPSWPTPP, two scenarios are considered: one is that the system is suitable for the logistics market; and the other is that the system is not suitable for the logistics market.

**Scenario 1**: When the system is adapted to the logistics market, the expected benefit is greater than the cost, i.e., *R*_1_ − *C*_1_ > 0, and *R*_2_ − *C*_2_ > 0. We discuss the following propositions (1, 2, 3, and 4).

**Proposition 1**: In the case of *F*_1_ + *G*_1_ − *C*_1_ > 0, *F*_2_ + *G*_2_ − *C*_2_ > 0 and *π* − *π*′ − *G*_1_ − *G*_2_ > 0, among the 8 pure equilibrium points (*E*_1_~*E*_8_) of the system, only *E*_8_(1,1,1) is the local asymptotic stability point. Thus, the ESS of the system will be (cooperation, cooperation, supervision).

**Proof of Proposition 1**: According to the eigenvalues of the Jacobian matrix corresponding to each equilibrium point in Table 3, we obtain the local stability corresponding to the equilibrium points of the system, as detailed in Table 4.

**Table 4:** Local stability analysis of equilibrium points (Proposition 1).

Equilibrium Point	*F*_1_ + *G*_1_ − *C*_1_ > 0, *F*_2_ + *G*_2_ − *C*_2_ > 0, *π* − (*π*′ + *G*_1_ + *G*_2_) > 0			
	λ_1_	λ_2_	λ_3_	Stability
***E***_**1**_ **(0,0,0)**	−	−	+	Saddle-point
***E***_**2**_ **(0,1,0)**	+	+	+	Nonstationary-point
***E***_**3**_ **(0,1,1)**	+	−	−	Saddle-point
***E***_**4**_ **(0,0,1)**	+	+	−	Saddle-point
***E***_**5**_ **(1,0,0)**	+	+	+	Nonstationary-point
***E***_**6**_ **(1,1,0)**	−	−	+	Saddle-point
***E***_**7**_ **(1,0,1)**	−	+	−	Saddle-point
***E***_**8**_ **(1,1,1)**	−	−	−	ESS

Proposition 1 shows that if the sum of the penalties for breach of contract and the bonuses for compliance with contract from the TPIP to the PPP and DC is greater than the respective costs of the PPP and DC, and the expected supervision profit of the TPIP is greater than the sum of the non-supervision profit and the bonus paid out, the rational players in the pooling system will choose the cooperation strategy, and opportunistic behavior will not occur.

Similarly, the conclusions of Propositions 2, 3, and 4 are as follows.

**Proposition 2**: If the sum of the penalties for breach of contract and the bonuses for compliance with contract from the TPIP to the PPP and DC is greater than the respective costs of the PPP and DC, but the expected supervision profit of the TPIP is less than the sum of the non-supervision profit and the bonus for the observant party, both the PPP and DC will choose the cooperation strategy, and the TPIP will select the non-supervision strategy. That is, in the case of *F*_1_ + *G*_1_ − *C*_1_ > 0, *F*_2_ + *G*_2_ − *C*_2_ > 0 and π′ − *G*_1_ − *G*_2_ < 0, only the equilibrium point *E*_6_(1,1,0) is the local asymptotically stable point.

**Proposition 3**: If the sum of the penalties for breach of contract and the bonuses for compliance with contract from the TPIP to the PPP and DC is less than the respective costs of the PPP and DC, and the expected supervision profit of the TPIP is greater than the sum of the non-supervision profit and the bonus for the observant party, the TPIP will select the supervision strategy, while both the PPP and DC will select either the betrayal strategy or the cooperation strategy, and the specific choice will be determined as per the initial conditions of the game-player. That is, in the case of *F*_1_ + *G*_1_ − *C*_1_ < 0, *F*_2_ + *G*_2_ − *C*_2_ < 0 and π − *π*′ − *G*_1_ − *G*_2_ > 0, only 2 equilibrium points, *E*_4_(0,0,1) and *E*_8_(1,1,1), are the local asymptotically stable points.

**Proposition 4**: If the sum of the penalties for breach of contract and the bonuses for compliance with contract from the TPIP to the PPP and DC is less than the respective costs of the PPP and DC, and the expected supervision profit of the TPP is less than the sum of the non-supervision profit and the bonus for the observant party, the TPIP will select the strategy of supervision when both the PPP and DC select the betrayal strategy; and if both the PPP and DC select the cooperation strategy, the TPIP will select the non-supervision strategy. That is, in the case of *F*_1_ + *G*_1_ − *C*_1_ < 0, *F*_2_ + *G*_2_ − *C*_2_ < 0 and π − *π*′ − *G*_1_ − *G*_2_ < 0, only 2 equilibrium points, *E*_4_(0,0,1) and *E*_6_(1,1,0), are the local asymptotically stable points.

**Scenario 2**: When the system is not adapted to the logistics market, the expected benefit is less than the cost, i.e., *R*_1_ − *C*_1_ < 0, and *R*_2_ − *C*_2_ < 0. We discuss the following propositions (5, 6, 7 and 8).

**Proposition 5**: In the case of *F*_1_ + *G*_1_ − *C*_1_ > 0, *F*_2_ + *G*_2_ − *C*_2_ > 0 and π − *π*′ − *G*_1_ − *G*_2_ > 0, among the 8 pure equilibrium points (*E*_1_~*E*_8_) of the system, only *E*_8_(1,1,1) is the local asymptotically stable point. Thus, the ESS of the system will be (cooperation, cooperation, supervision).

**Proof of Proposition 5**: According to the eigenvalues of Jacobian matrix in Table 2, we obtain the local stability analysis results of the equilibrium points *E*_1_~*E*_8_ of the pooling system, as shown in Table 5.

**Table 5:** Local stability analysis of equilibrium points (Proposition 5).

Equilibrium Point	*F*_1_ + *G*_1_ − *C*_1_ > 0, *F*_2_ + *G*_2_ − *C*_2_ > 0, *π* − (*π*′ + *G*_1_ + *G*_2_) > 0			
	λ_1_	λ_2_	λ_3_	Stability
***E***_**1**_ **(0,0,0)**	−	−	+	Saddle-point
***E***_**2**_ **(0,1,0)**	−	+	+	Saddle-point
***E***_**3**_ **(0,1,1)**	+	−	−	Saddle-point
***E***_**4**_ **(0,0,1)**	+	+	−	Saddle-point
***E***_**5**_ **(1,0,0)**	+	−	+	Saddle-point
***E***_**6**_ **(1,1,0)**	+	+	+	Nonstationary-point
***E***_**7**_ **(1,0,1)**	−	+	−	Saddle-point
***E***_**8**_ **(1,1,1)**	−	−	−	ESS

Proposition 5 shows that although the pallet pooling system cannot be effectively adapted to the current logistics market, if the sum of the penalties for breach of contract and the bonuses for compliance with contract from the TPIP to the PPP and DC is greater than the respective costs of the PPP and DC, and the expected supervision profit of the TPIP is greater than the sum of the non-supervision profit and the bonus for the observant party, the rational players of the pooling system will adopt the cooperation strategy, and opportunistic behavior will not occur.

Similarly, we obtain the conclusions of Propositions 6, 7, and 8 as follows.

**Proposition 6**: In scenario 2, if the sum of the penalties for breach of contract and the bonuses for compliance with contract from the TPIP to the PPP and DC is greater than the respective costs of the PPP and DC, but the expected supervision profit of the TPIP is less than the sum of the non-supervision profit and the bonus for the observant party, the system will be unstable, and cannot operate normally. That is, in the case of *F*_1_ + *G*_1_ − *C*_1_ > 0, *F*_2_ + *G*_2_ − *C*_2_ > 0 and π − *π*′ − *G*_1_ − *G*_2_ < 0, no local asymptotically stable point exists among the 8 equilibrium points (*E*_1_~*E*_8_) of the system.

**Proposition 7**: In scenario 2, if the sum of the penalties for breach of contract and the bonuses for compliance with contract from the TPIP to the PPP and DC is less than the respective costs of the PPP and DC, and the expected supervision profit of the TPIP is greater than the sum of the non-supervision profit and the bonus for the observant party, there are two cases: (1) if the sum of the penalties for breach of contract and the bonuses for compliance with contract from the TPIP to the PPP and DC is greater than the difference between the respective costs and profits of the PPP and DC, the TPIP will choose the supervision strategy, while both the PPP and DC will choose either the betrayal strategy or the cooperation strategy, and the specific strategy to be selected depends on the initial conditions of the game-players; (2) if the sum of the penalties for breach of contract and the bonuses for compliance with contract from the TPIP to the PPP and DC is less than the difference between the respective costs and the profits of the PPP and DC, both the PPP and DC will choose the betrayal strategy, and the TPIP will choose the supervision strategy. That means that in the case of *F*_1_ + *G*_1_ − *C*_1_ < 0, *F*_2_ + *G*_2_ − *C*_2_ < 0 and π − *π*′ − *G*_1_ − *G*_2_ > 0: (1) if *C*_1_ − *R*_1_ < *F*_1_ + *G*_1_ < *C*_1_ and *C*_2_ − *R*_2_ < *F*_2_ + *G*_2_ < *C*_2_, two equilibrium points E_4_(0,0,1) and *E*_8_(1,1,1) are the local asymptotically stable points; (2) if *F*_1_ + *G*_1_ < *C*_1_ − *R*_1_ and *F*_2_ + *G*_2_ < *C*_2_ − *R*_2_, only the equilibrium point E_4_(0,0,1) is the local asymptotically stable point.

**Proposition 8**: In scenario 2, if the sum of the penalties for breach of contract and the bonuses for compliance with contract from the TPIP to the PPP and DC is less than the respective costs of the PPP and DC, and the expected supervision profit of the TPIP is less than the sum of the non-supervision profit and the bonus for the observant party, the PPP and DC will select the betrayal strategy, and the TPIP will select the supervision strategy. That is, in the case of *F*_1_ + *G*_1_ − *C*_1_ < 0, *F*_2_ + *G*_2_ − *C*_2_ < 0 and π − *π*′ − *G*_1_ − *G*_2_ < 0, only the equilibrium point *E*_1_(0,0,1) among eight pure equilibrium points *E*_1_~*E*_8_ of the system is the local asymptotically stable point.

## 5. Numerical simulation analysis

### 5.1. Simulation parameter settings

From the above analysis, the PPSWPTPP is a tripartite evolutionary game system. The game strategies of all the players can be divided into two categories, pure strategy and mixed strategy, with three pure strategies and two mixed strategies included. The evolution results of all the propositions are shown in Table 6.

**Table 6:** Evolution results of each proposition in tripartite game.

Serial Number		Stable Point	ESS	Proposition
**Pure Strategy**	**1**	(1,1,1)	(cooperation, cooperation, supervision)	1; 5
	**2**	(1,1,0)	(cooperation, cooperation and non-supervision)	2
	**3**	(0,0,1)	(betrayal, betrayal and supervision)	7(2); 8
**Mixed Strategy**	**4**	(0,0,1) or (1,1,1)	(betrayal, betrayal and supervision) or (cooperation, cooperation, supervision)	3; 7(1)
	**5**	(0,0,1) or (1,1,0)	(betrayal, betrayal and supervision) or (cooperation, cooperation and non-supervision)	4

**Fig 5:**
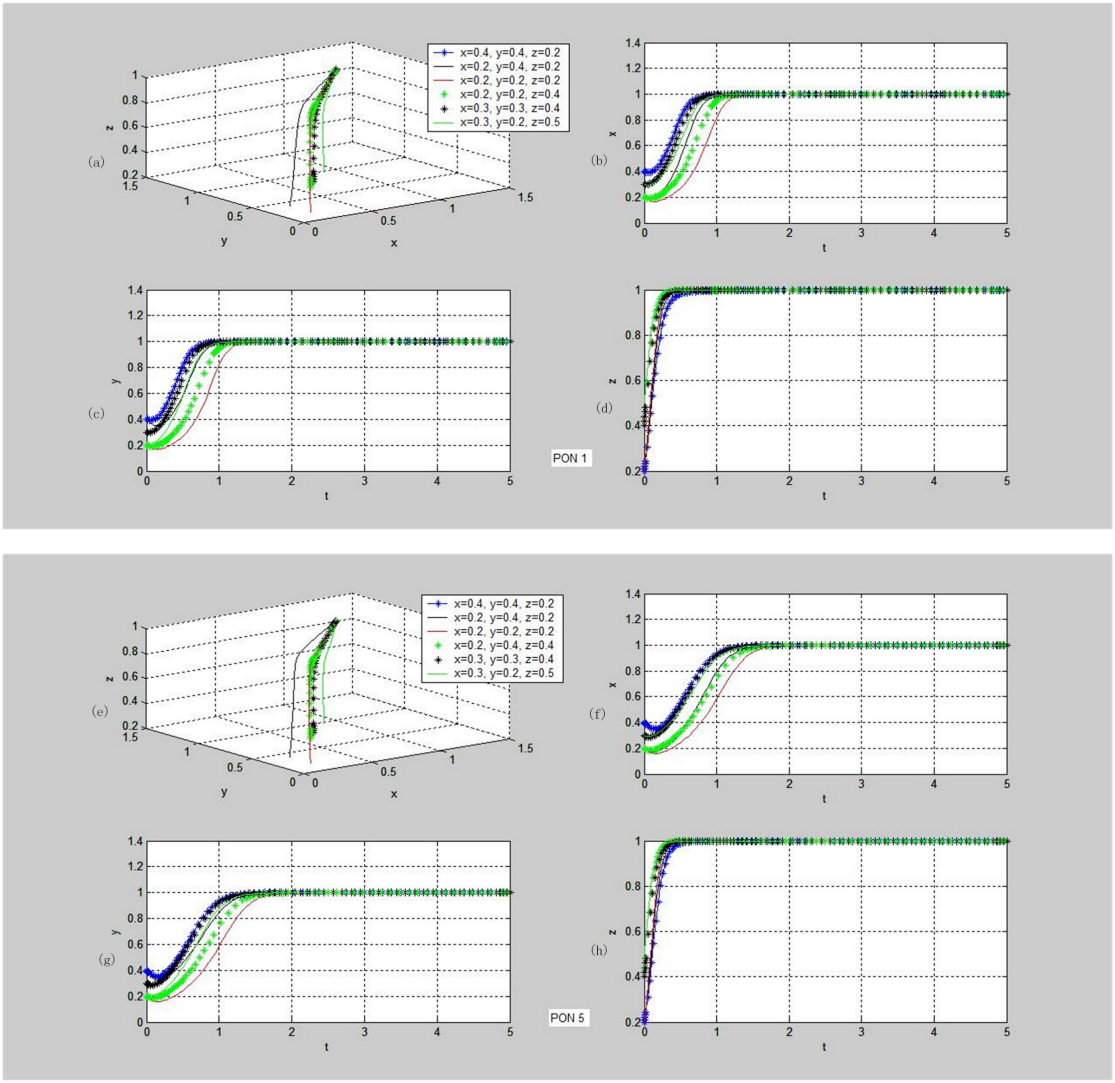
Evolutionary dynamics of strategies stable in (cooperation, cooperation, supervision). PON-Proposition. (a)~(d) demonstrate the phase trajectories of x, y, z and (x, y, z) in the game situations shown in Proposition 1: (a) The phase trajectory of (x, y, z) of the entire evolutionary game system over time t; (b) the dynamic evolution characteristic of x, i.e., the proportion of individuals choosing Cooperation strategy in population PPP over time t; (c) the dynamic evolution characteristic of y, i.e., the proportion of individuals choosing Cooperation strategy in population DC over time t; (d) the dynamic evolution characteristic of z, i.e., the proportion of individuals choosing Supervision strategy in population TPIP over time t. (e)~(h) demonstrate the phase trajectories of x, y, z and (x, y, z) in the game situations shown in Proposition 5: (e) The phase trajectory of (x, y, z) of the entire evolutionary game system over time t; (f) the dynamic evolution characteristic of x, i.e., the proportion of individuals choosing Cooperation strategy in population PPP over time t; (g) the dynamic evolution characteristic of y, i.e., the proportion of individuals choosing Cooperation strategy in population DC over time t; (h) the dynamic evolution characteristic of z, i.e., the proportion of individuals choosing Supervision strategy in population TPIP over time t.

**Fig 6:**
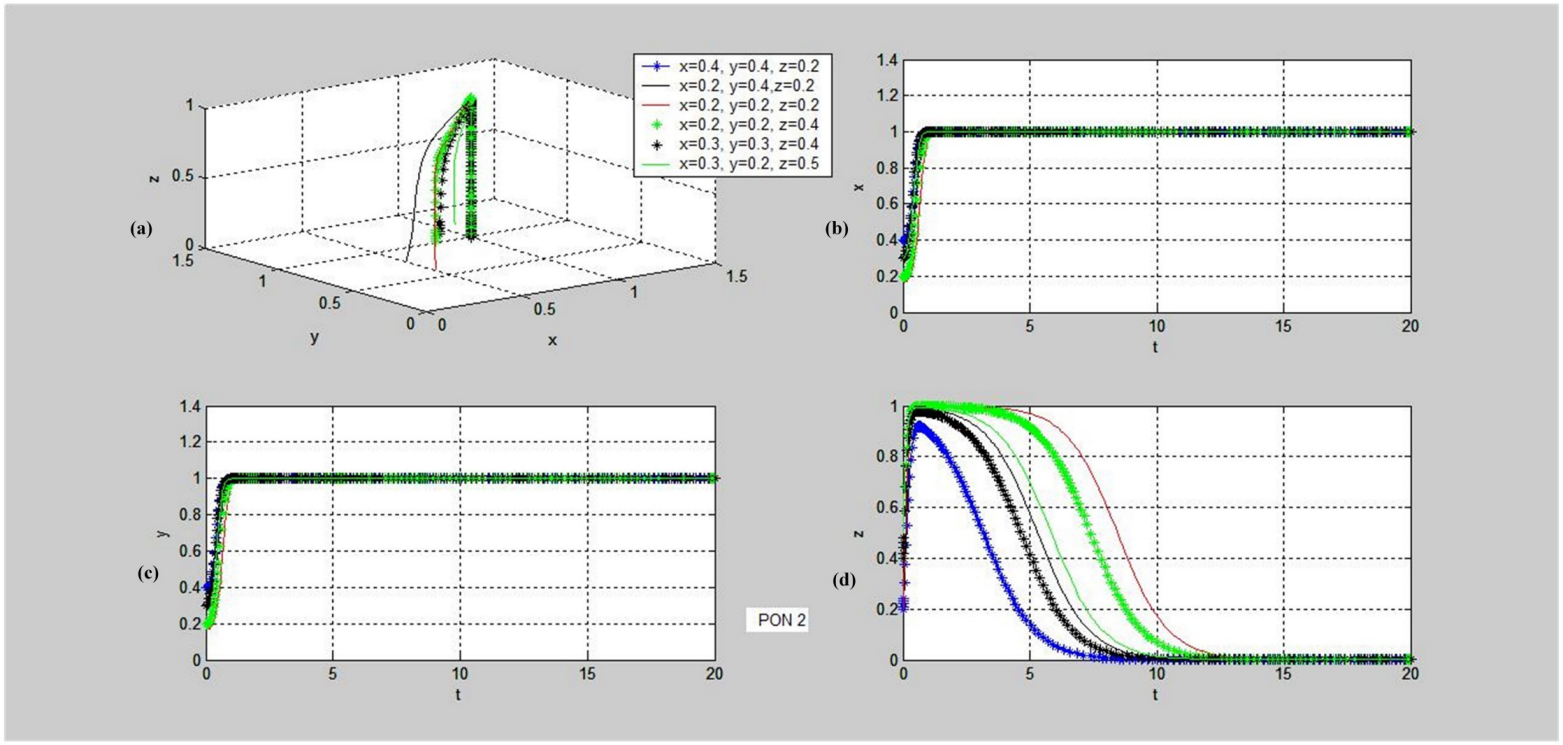
Evolutionary dynamics of strategies stable in (cooperation, cooperation, non-supervision). PON-Proposition. (a)~(d) demonstrate the phase trajectories of x, y, z and (x, y, z) in the game situations shown in Proposition 2: (a) The phase trajectory of (x, y, z) of the entire evolutionary game system over time t; (b) the dynamic evolution characteristic of x, i.e., the proportion of individuals choosing Cooperation strategy in population PPP over time t; (c) the dynamic evolution characteristic of y, i.e., the proportion of individuals choosing Cooperation strategy in population DC over time t; (d) the dynamic evolution characteristic of z, i.e., the proportion of individuals choosing Non-supervision strategy in population TPIP over time t.

**Fig 7:**
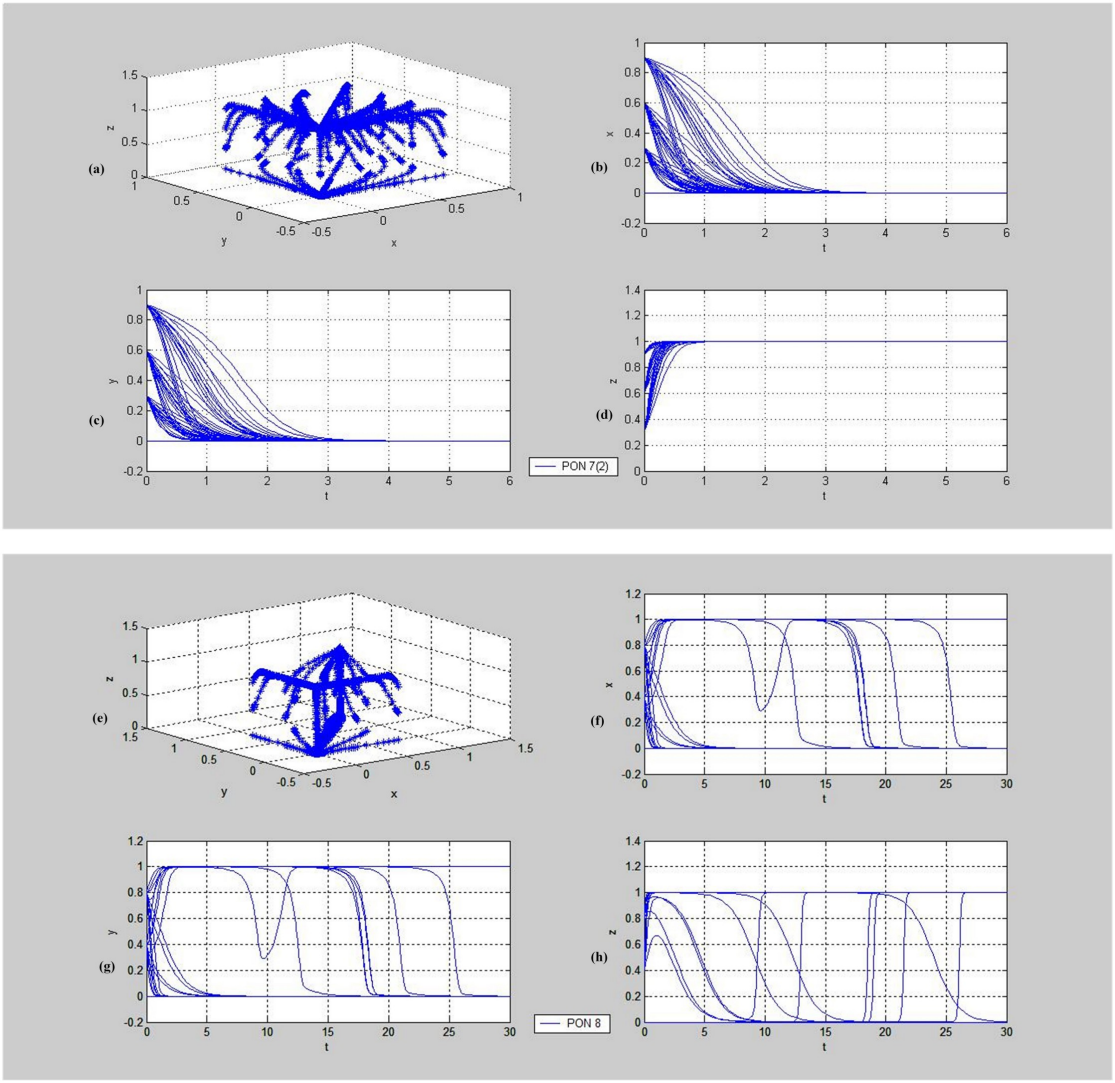
Evolutionary dynamics of strategies stable in (betrayal, betrayal, supervision). PON-Proposition. (a)~(d) demonstrate the phase trajectories of x, y, z and (x, y, z) in the game situations shown in Proposition 7(2): (a) The phase trajectory of (x, y, z) of the entire evolutionary game system over time t; (b) the dynamic evolution characteristic of x, i.e., the proportion of individuals choosing Cooperation strategy in population PPP over time t; (c) the dynamic evolution characteristic of y, i.e., the proportion of individuals choosing Cooperation strategy in population DC over time t; (d) the dynamic evolution characteristic of z, i.e., the proportion of individuals choosing Supervision strategy in population TPIP over time t. (e)~(h) demonstrate the phase trajectories of x, y, z and (x, y, z) in the game situations shown in Proposition 8: (e) The phase trajectory of (x, y, z) of the entire evolutionary game system over time t; (f) the dynamic evolution characteristic of x, i.e., the proportion of individuals choosing Cooperation strategy in population PPP over time t; (g) the dynamic evolution characteristic of y, i.e., the proportion of individuals choosing Cooperation strategy in population DC over time t; (h) the dynamic evolution characteristic of z, i.e., the proportion of individuals choosing Supervision strategy in population TPIP over time t.

**Fig 8:**
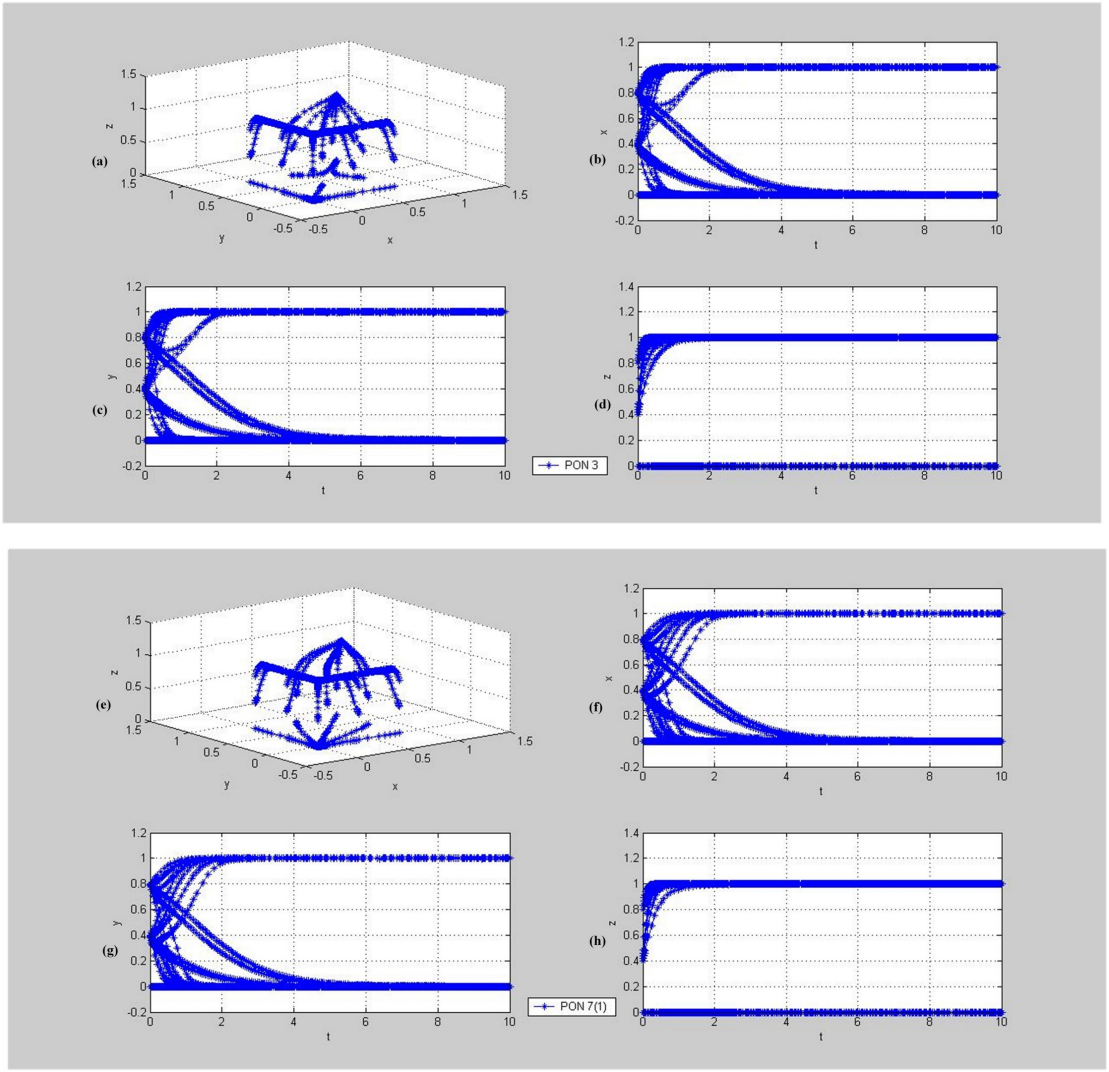
Evolutionary dynamics of mixed strategies (cooperation, cooperation, supervision) or (betrayal, betrayal, supervision). PON-Proposition. (a)~(d) demonstrate the phase trajectories of x, y, z and (x, y, z) in the game situations shown in Proposition 3: (a) The phase trajectory of (x, y, z) of the entire evolutionary game system over time t; (b) the dynamic evolution characteristic of x, i.e., the proportion of individuals choosing Cooperation strategy in population PPP over time t; (c) the dynamic evolution characteristic of y, i.e., the proportion of individuals choosing Cooperation strategy in population DC over time t; (d) the dynamic evolution characteristic of z, i.e., the proportion of individuals choosing Supervision strategy in population TPIP over time t. (e)~(h) demonstrate the phase trajectories of x, y, z and (x, y, z) in the game situations shown in Proposition 7(1): (e) The phase trajectory of (x, y, z) of the entire evolutionary game system over time t; (f) the dynamic evolution characteristic of x, i.e., the proportion of individuals choosing Cooperation strategy in population PPP over time t; (g) the dynamic evolution characteristic of y, i.e., the proportion of individuals choosing Cooperation strategy in population DC over time t; (h) the dynamic evolution characteristic of z, i.e., the proportion of individuals choosing Supervision strategy in population TPIP over time t.

**Fig 9:**
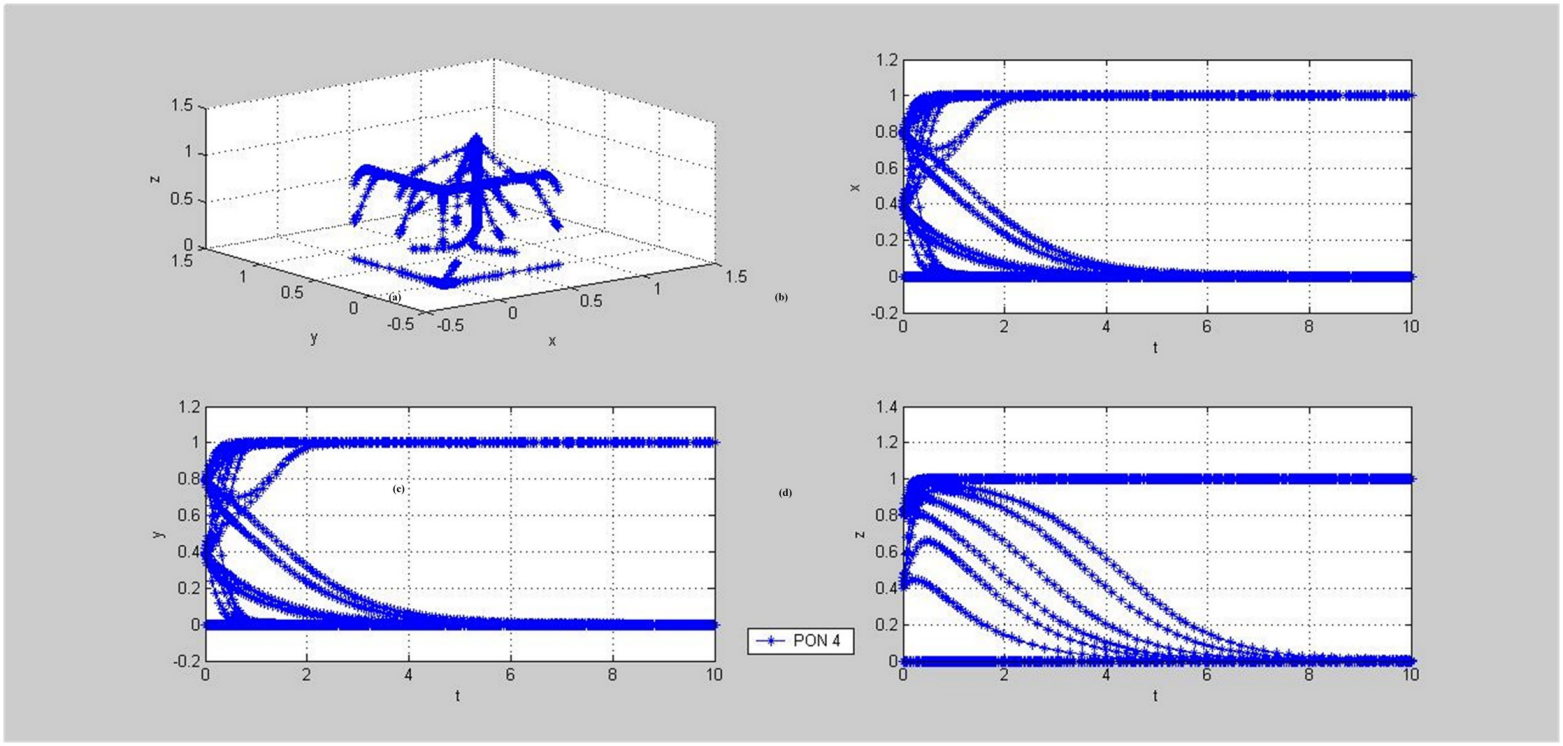
Evolutionary dynamics of mixed strategies (cooperation, cooperation, non-supervision) or (betrayal, betrayal, supervision). PON-Proposition. (a)~(d) demonstrate the phase trajectories of x, y, z and (x, y, z) in the game situations shown in Proposition 4: (a) The phase trajectory of (x, y, z) of the entire evolutionary game system over time t; (b) the dynamic evolution characteristic of x, i.e., the proportion of individuals choosing Cooperation strategy in population PPP over time t; (c) the dynamic evolution characteristic of y, i.e., the proportion of individuals choosing Cooperation strategy in population DC over time t; (d) the dynamic evolution characteristic of z, i.e., the proportion of individuals choosing Non-supervision strategy in population TPIP over time t.

To further explore the process of evolution from the initial point to the stable point in the tripartite-game system, we used MATLAB numerical simulations to verify the theoretical analysis results of the phase evolution mode and evolution stability of three different pure strategies and two different mixed strategies, in the case of 0 ≤ x ≤ 10 ≤ y ≤ 1 and 0 ≤ z ≤ 1. In the following Figs 5–9, Axis x indicates the intention degree of the PPP to choose the cooperation strategy; Axis y shows the intention degree of the DC to choose the cooperation strategy; and Axis z reflects the intention degree of the TPIP to choose the supervision strategy. Based on the conditions in the propositions, we set the evolutionary game parameters of all the propositions for the PPSWPTPP, as shown in Table 7, and analyzed the simulation results of all the propositions.

**Table 7:** Parameters of evolutionary game for each proposition of the PPSWPTPP.

Proposition	π	π′	*R*_1_	*R*_2_	*C*_1_	*C*_2_	*G*_1_	*G*_2_	*F*_1_	*F*_2_	*S*_1_	*S*_2_	ESS
**1**	11	4	10	10	6	6	3	3	4	4	2	2	(cooperation, cooperation, supervision)
**2**	11	6	10	10	6	6	3	3	4	4	2	2	(cooperation, cooperation and non-supervision)
**3**	11	4	10	10	6	6	2	2	3	3	2	2	Mixed Strategy 4
**4**	11	8	10	10	6	6	2	2	3	3	2	2	Mixed Strategy 5
**5**	11	4	5	5	6	6	3	3	4	4	2	2	(cooperation, cooperation, supervision)
**7(1)**	11	5	5	5	6	6	2	2	3	3	2	2	Mixed Strategy 4
**7(2)**	11	5	2	2	6	6	1	1	2	2	2	2	(betrayal, betrayal and supervision)
**8**	11	8	5	5	6	6	2	2	3	3	2	2	(betrayal, betrayal and supervision)

### 5.2. Evolution dynamics of all propositions in case of pure strategy

In the PPSWPTPP, the game player’s selection of the pure strategy means that the PPP, DC and TPIP either choose the strategy of pooling (cooperation) and supervision or select the strategy of non-pooling (betrayal) and non-supervision. In the results of the theoretical analysis, there are three pure strategy profiles, including (cooperation, cooperation, supervision), (cooperation, cooperation and non-supervision) and (betrayal, betrayal and supervision).

#### 5.2.1. Dynamic evolution of (cooperation, cooperation, supervision) and (cooperation, cooperation, non-supervision)

To observe the evolution trend from the initial point to the stable point and retain generality, 6 representative initial observation values, (0.4,0.4,0.2), (0.2,0.4,0.2), (0.2,0.2,0.2), (0.2,0.2,0.4), (0.3,0.3,0.4), and (0.3,0.2,0.5), are selected for data simulation, as indicated in Fig 5.

The subgraphs in Fig 5 are the system dynamic evolution diagrams drawn through the computer simulation on all the propositions in Table 7 evolved to the strategy of (cooperation, cooperation and supervision) as per the set parameters. The system will eventually evolve to the stable point (1,1,1) regardless of the initial values of x, y and z, but the evolution paths are slightly different. For example, in terms of the rate of rising from the Value *x* and Value *y* of the initial point to the evolutionary stable point (1,1,1), the rate in Proposition 1 is lower than that of the Value z, while the rate of the Value z in Proposition 5 is higher. The simulation results verify the conclusions of all the propositions, showing that the system can be implemented smoothly.

Similarly, we obtain the simulation results of Proposition 2, as shown in Fig 6. Although the values tend to increase at first from Value *x*, Value *y* and Value z of the initial point, they all evolve to the stable point (1,1,0) eventually. Under this condition, the trilateral pallet pooling system cannot be carried out smoothly, but the pallet pooling can be conducted between the supplier and the demander.

#### 5.2.2. Dynamic evolution of (betrayal, betrayal, supervision)

In order to make the evolution dynamics richer and more intuitive, the initial values of *x*, y and z are set in the range of [0,1] for simulation, and the step size is 0.2, as shown in Fig 7. All the initial points will eventually be stabilized at the point (0,0,1) through the system evolution, while the evolution paths of Propositions 7(2) and 8 are different: for Proposition 7(2), Value z first evolves to 1 rapidly, and then Values *x* and *y* suddenly drop to 0; the evolution path of Proposition 8 is more complex than that of Proposition 7(2), and when the initial values of *x* and *y* are small, the evolution path of Proposition 8 is similar to that of Proposition 7(2); when the initial values of *x* and *y* are large, the system tends to evolve towards the point (1,1,1), but eventually it evolves to the point (0,0,1). In this case, the system cannot work smoothly because of betrayal of both the supplier and demander.

### 5.3. Evolution dynamics of all propositions in case of mixed strategy

Mixed strategy means that the strategy selected by the players is not unique but given by a probability distribution in the strategy space. From Table 6, in the PPSWPTPP, there are two mixed strategies. One is (betrayal, betrayal, supervision) or (cooperation, cooperation, supervision) under the conditions in Propositions 3 and 7(1), and the other is (betrayal, betrayal, supervision) or (cooperation, cooperation, non-supervision) under the conditions in Propositions 4.

Proposition 3 has three conditions. Firstly, the system adapted to the logistics market (i.e., Scenario 1). Secondly, the expected supervision profit of the TPIP is greater than the sum of the non-supervision profit and the bonus paid out; and thirdly, the sum of the penalties for breach of contract and the bonuses for compliance with contract from the TPIP to the PPP and DC is less than the respective costs of the PPP and DC. There are also three conditions for Propositions 7(1). Firstly, the system is not adapted to the current logistics market (i.e., Scenario 2). Secondly, the sum of the penalties for breach of contract and the bonuses for compliance with contract from the TPIP to the PPP and DC is less than the respective costs of the PPP and DC. Thirdly, the sum of the penalties for breach of contract and the bonuses for compliance with contract from the TPIP to the PPP and DC is greater than the difference between the respective costs and the profits of the PPP and DC. Under the conditions in Propositions 3 and 7(1), the players of the system may make a decision of cooperation. However, which strategy the system will ultimately choose is determined by the initial intention of the PPP and DC, that is, the initial values of x and y, which means that the intention degree for cooperation of the pallet supplier and demander plays a decisive role in the smooth development of the system.

In case that the system is adapted to the logistics market, if the expected supervision profit of the TPIP is less than the sum of the non-supervision profit and the bonus paid out, the system may evolve to the situation in which both the PPP and DC choose to cooperate while the TPIP may choose the non-supervision strategy under the condition in Proposition 4. In this case, the tripartite system cannot be carried out smoothly, but the pallet pooling can be carried out between the supply and demand sides. The initial values of *x*, y and z selected for simulation here are the same as above, that is, the value range is [0,1] and the step size is 0.2, as shown in Figs 8 and 9.

#### 5.3.1. Dynamic evolution of (betrayal, betrayal, supervision) or (cooperation, cooperation, supervision)

As shown in Fig 8, in the case of Propositions 3 and 7(1), the system may eventually evolve into two different equilibrium points, namely (0,0,1) or (1,1,1). The PPP and DC choose the same strategy, either betrayal or cooperation, while the TPIP only chooses the supervision strategy. From the subgraphs in Fig 8, we found that if the values of *x* and *y* are large in the initial Values *x*, y and z, the system will evolve to the point (1,1,1), otherwise it will evolve to the point (0,0,1). In other words, in the case of Propositions 3 and 7(1), the smooth operation of the system requires the high initial participation intention of the PPP and DC, which indicates that the TPIP should try to select PPPs and DCs with greater intention and confidence for pallet pooling as the cooperative partners.

#### 5.3.2. Dynamic evolution of (betrayal, betrayal, supervision) or (cooperation, cooperation, non-supervision)

As shown in Fig 9, in the case of Proposition 4, the system may eventually evolve into two different equilibrium points, namely (1,1,0) or (0,0,0). The PPP and DC will choose the same strategy, either cooperation or betrayal; when the PPP and DC adopt the cooperation strategy, the TPIP will adopt the non-supervision strategy, and vice versa, verifying the correctness of the theoretical analysis.

## 6. Conclusion and discussion

The PPWPTPP is one of the pilot modes of pallet pooling in China. The TPIP as the intermediary connects the pallet supplier and demander, forming a three-in-one pallet operation ecosystem, and they depend on and influence each other. Based on the evolutionary game model, this study sets eight propositions in two scenarios regarding whether pallet pooling is adapted to the logistics market, to study the stability and dynamic evolution process of the players in the PPSWPTPP, and to analyze the impact of the penalty mechanism for breach of contract on the strategy selection in the system. We obtain the relevant scenarios and conditions for smooth implementation of the PPSWPTPP, as shown in Propositions 1, 2, 3, 5, and 7(1) in Table 6.

The theoretical analysis is summarized and discussed below, and relevant suggestions are provided.

### (1) Reasonable setting of penalties and bonuses in the PPSWPTPP can ensure the smooth development of pallet pooling.

In the tripartite-game of PPSWPTPP, no matter whether the pallet pooling adapts to the logistics market, when the sum of the penalties for breach of contract and the bonuses for compliance with contract from the TPIP to the PPP and DC is greater than their respective costs, and the expected supervision profit of the TPIP is greater than the sum of the non-supervision profit and the bonuses paid, rational players will choose the cooperation strategy without opportunistic behavior, shown in the conclusions of Proposition 1 in scenario 1 and Proposition 5 in scenario 2. In other words, if the amount of bonuses and penalties set by the TPIP is relatively low, it is difficult to mobilize the enthusiasm of the pallet suppliers and demanders, resulting in the difficult implementation of the pooling. By contrast, if the amount of bonus is too large, the TPIP will have difficulty bearing the amount of bonus paid, and ultimately choose the non-supervision strategy. In either case, the pallet pooling cannot run smoothly. Thus, we suggest that the TPIP should conduct a full investigation before issuing the policies of reward and punishment, to collect relevant information and accurately assess the possible risks brought by the violation of the suppliers and demanders and the amount of bonus that the platform can bear. We should not only mobilize the enthusiasm of the suppliers and demanders, but also control the own risks of the TPIP to ensure the smooth implementation of pallet pooling.

### (2) Reasonable and scientific estimation of the pooling benefits has a decisive role for the suppliers and demanders to adopt the cooperation strategy.

As long as the expected benefits of the supplier and demander participating in the pooling is greater than the input costs, even if the sum of the penalties and bonuses imposed by the TPIP on the PPP and DC is less than the pooling input costs, the ultimate evolution strategy of the supplier and demander may be cooperation. The probability of cooperation is related to the initial intention, while the initial intention is related to the expected benefits. For example, in the case where the initial intention degree of the DC is equal to or greater than [*C*_1_ − (*G*_1_ + *F*_1_)z]/*R*_1_, the PPP will choose the cooperation strategy. When the initial intention degree of the PPP is equal to or greater than [*C*_2_ − (*G*_2_ + *F*_2_)z]/*R*_2_, the DC will eventually choose the cooperation strategy.

Thus, before attracting suppliers and demanders to participate in pooling, the TPIP should set scientific and reasonable objectives, and fully consider various risks undertaken by the players and the benefit allocation, which can make up for the failure of the pooling system due to insufficient estimation of liquidated damages, penalties and bonuses.

### (3) The key factor for the smooth implementation of the PPSWPTPP is the strong intention of the players to the pallet pooling.

If the sum of the penalties for breach of contract and the bonuses for compliance with contract from the TPIP to the PPP and DC is less than their respective costs, it does not mean that pallet pooling will fail. In this case, if the expected pooling profit of the TPIP is considerable, two evolution results of the system may exist; that is, both the PPP and DC select the strategy of betrayal or cooperation, and the specific evolution of the strategy is related to the initial intention degree of the players to cooperate (i.e., probability of adopting the strategy of cooperation), as shown in the conclusions of Propositions 3 and 7(1). The closer to 1 the initial values of the intention degree *x* of the PPP and the intention degree *y* of the DC, the shorter the time for the pooling system to evolve into the strategy of (cooperation, cooperation, supervision). Thus, we suggest that the TPIP attract pallet suppliers and demanders that have successful experience in pooling to join the platform when selecting cooperative partners, because previous experience is beneficial to improving the confidence of the cooperators. Even if there are few penalties and bonuses set in the platform, mutual trust among the players can reduce the probability of default and promote the success of the pooling.

### (4) Pallet pooling is closely related to logistics market demand, and a stable pooling system must match the market.

As shown in Table 6, a total of five propositions exist where pallet pooling can be implemented smoothly, including three propositions of pure strategy and two propositions of mixed strategy. Only Proposition 5 among the propositions of pure strategy and Proposition 7(1) among the propositions of mixed strategy are obtained in the scenario that pallet pooling is not adapted to the logistics market, and the remaining three propositions in the scenario relate to pallet pooling being adapted to the logistics market, showing that pallet pooling is closely related to the logistics market demand, and the probability of stability of the pooling system in case of adaptation to the logistics market is high. Therefore, pallet pooling activities shall not be separated from the logistics market demand. This also explains why several early attempts for pallet pooling in China ended in failure (such as the pilot project in 1965).

Based on this study, several future research directions may be undertaken. For instance, empirical research on pallet pooling in China needs to be carried out. As an additional further research direction, the model developed in this paper could be redesigned to consider parameters such as transaction cost and operation efficiency.
